# RNA sequencing reveals metabolic and regulatory changes leading to more robust fermentation performance during short-term adaptation of *Saccharomyces cerevisiae* to lignocellulosic inhibitors

**DOI:** 10.1186/s13068-021-02049-y

**Published:** 2021-10-15

**Authors:** Marlous van Dijk, Peter Rugbjerg, Yvonne Nygård, Lisbeth Olsson

**Affiliations:** Department of Biology and Bioengineering, Division of Industrial Biotechnology, Chalmers University of Technology, Kemivägen 10, 412 96 Gothenburg, Sweden

**Keywords:** Short-term adaptation, Inhibitor stress, Transcriptomics, YHK8, Industrial yeast strain

## Abstract

**Background:**

The limited tolerance of *Saccharomyces cerevisiae* to inhibitors is a major challenge in second-generation bioethanol production, and our understanding of the molecular mechanisms providing tolerance to inhibitor-rich lignocellulosic hydrolysates is incomplete. Short-term adaptation of the yeast in the presence of dilute hydrolysate can improve its robustness and productivity during subsequent fermentation.

**Results:**

We utilized RNA sequencing to investigate differential gene expression in the industrial yeast strain CR01 during short-term adaptation, mimicking industrial conditions for cell propagation. In this first transcriptomic study of short-term adaption of *S. cerevisiae* to lignocellulosic hydrolysate, we found that cultures respond by fine-tuned up- and down-regulation of a subset of general stress response genes. Furthermore, time-resolved RNA sequencing allowed for identification of genes that were differentially expressed at 2 or more sampling points, revealing the importance of oxidative stress response, thiamin and biotin biosynthesis. furan-aldehyde reductases and specific drug:H^+^ antiporters, as well as the down-regulation of certain transporter genes*.*

**Conclusions:**

These findings provide a better understanding of the molecular mechanisms governing short-term adaptation of *S. cerevisiae* to lignocellulosic hydrolysate, and suggest new genetic targets for improving fermentation robustness.

**Supplementary Information:**

The online version contains supplementary material available at 10.1186/s13068-021-02049-y.

## Background

The use of lignocellulosic material as a substrate for the production of renewable fuels is an attractive alternative to petrochemical-based processes and high carbon-emission industries. However, lignocellulosic hydrolysates are notoriously inhibitory to microorganisms due to the pretreatment required to release the monomeric sugars from the raw materials [1–3]. In various attempts to remedy this, industrial strains of *Saccharomyces cerevisiae* have been genetically engineered or subjected to adaptive evolution to improve their tolerance (e.g., [4–7]). However, inhibitor stress is still a significant problem in second-generation bioethanol production.

Short-term adaptation has been shown to improve inhibitor tolerance, and thus ethanol productivity [8–11]. Short-term adaptation can be achieved by adding a dilute solution of the hydrolysate to the medium during cultivation (propagation) prior to fermentation. However, the molecular mechanisms behind improved robustness remain unexplored. In the bioethanol industry, aerobic propagation is used to produce cell mass to inoculate anaerobic fermentations where growth does not usually exceed two doublings, and cell titers are directly correlated to ethanol productivity [12, 13]. Ethanol production from lignocellulosic hydrolysates is limited by the high abundance of inhibitors such as furfurals, weak acids and phenolics. Detoxification of furfural is important, and is usually achieved by NADPH-requiring oxidoreductases (e.g., aldehyde reductase Ari1 and the methylglyoxal reductases Gre2 and Gre), or alcohol dehydrogenases (Adh1, Adh6, and Adh7), all of which are up-regulated under furfural stress [14, 15]. Furfural generates high intracellular concentrations of reactive oxygen species (ROS). ROS are thought to be involved in the decrease in cell viability, and thus the lower ethanol productivity at elevated ethanol concentrations [16]. In a parallel or additional strategy to short-term adaptation, the addition of antioxidants such as the B vitamins thiamin and biotin, and the antioxidant glutathione has been shown to improve ethanol productivity in hydrolysate fermentation [5, 17, 18]. These different antioxidants are believed to act as sinks for ROS or regenerators of NADPH through various mechanisms, thereby relieving inhibitor stress.

Studies on the transcriptomic response of *S. cerevisiae* to inhibitor stress have mainly focused on either single inhibitors, such as acetic acid [19–21], furfural [21–23] or hydroxymethylfurfural (HMF) [20, 24, 25], or synthetic mixtures of inhibitors [7, 26–28]. However, the response of *S. cerevisiae* to a cocktail of inhibitors is markedly different from that to individual compounds, due to synergistic effects [26]. To the best of the authors’ knowledge, no transcriptomics studies have been carried out on yeast propagation in the presence of real lignocellulosic hydrolysates [2, 26], however, such studies are important to improve the efficiency of industrial applications.

*S. cerevisiae* strains harboring recombinant xylose assimilation pathways based on xylose reductase and xylitol dehydrogenase are commonly used in conversion of lignocellulose-derived streams to improve overall yields [29, 30]. The heterologously converted D-xylulose 5-phosphate enters the native metabolism by conversion of the transketolases Tkl1 and Tkl2 to D-glyceraldehyde 3-phosphate. In this study, we have investigated the transcriptional response of the industrial xylose-consuming S. cerevisiae strain CR01 to short-term adaptation in lignocellulosic hydrolysate using a fed-batch propagation scheme. The used lignocellulose hydrolysate was a steam-pretreated wheat straw. The specific aim of this study was to investigate the transcriptome in order to identify cellular strategies in the short-term adaptive response to hydrolysate that may lead to improved robustness and ethanol productivity in subsequent hydrolysate fermentation. The knowledge gained will provide valuable information on suitable optimization targets for the further improvement of yeast performance. Obtaining reproducible samples for RNA sequencing is challenging due to the complexity of the lignocellulosic substrates as well as batch variation in hydrolysate composition. Furthermore, fed-batch propagation as used in industry is a dynamic process starting with batch phase, followed by at feeding phase. When comparing short-term adaptation to no adaptation, we used the same feeding rate in the feed phase to allow for as comparable growth conditions as possible. Samples were taken during the time course of the feeding during which the hydrolysate concentration was increased exponentially by feeding in the adapting culture. Data-driven analysis was used to determine transcriptional trends during short-term adaptation. We identified and studied changes in general stress response, changes in the expression of transcription factors and in metabolic pathways related to nutritional factors.

## Results and discussion

When designing the RNA-seq data collection, we carefully considered the set-up and alternative set-ups, as one should strive for comparing RNA-seq datasets under conditions that allow comparison only varying in the parameter under investigation. Here, the non-adopting cultures exhibited respiratory metabolism under the feed phase, whereas the adopting cultures exhibited respiratory–fermentative metabolism under the feed phase. We have investigated the experimental regime in detail, and it has not been possible to find conditions where the adopting culture exhibited respiratory metabolism during the full feeding phase in a reproducible manner. The consistency of lignocellulosic hydrolysates, low feeding rates and volumes is difficult to supply in a reproducible manner. Consequently, our experiments were performed under identical conditions for the adapting and non-adapting cultures, resulting in difference in physiological behavior.

### Cellular physiology during and after short-term adaptation

In order to investigate the transcriptomic response of *S. cerevisiae* strain CR01 during short-term adaptation to wheat straw hydrolysate (WSH), fed-batch cultures were propagated with (adapting) and without (non-adapting) 40% (w/w) WSH in the feed solution. The fed-batch cultures started with a 13.5-h-long batch phase, after which a 28-h feed was started. The applied feed rate was the same in the adapting and non-adapting cultures. The propagation set-up was chosen to mimic industrial conditions for cell mass production, while maintaining controlled conditions. During the feeding phase, ethanol produced in the batch phase may be reassimilated and sugars (such as sucrose, glucose and xylose) will be continuously used as carbon and energy source and consequently their concentration in the culture broth is low.

**Fig. 1:**
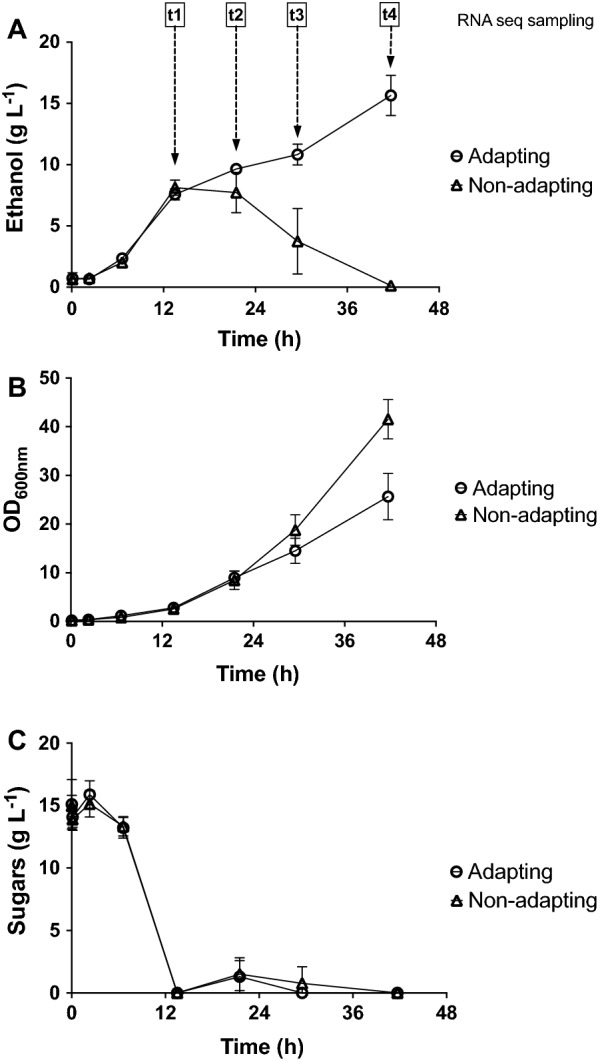
Propagation performance of adapting (open circles, 40% (w/w) hydrolysate-fed) and non-adapting (open triangles) aerobic *S. cerevisiae* CR01 cultures: **A** ethanol concentration, **B** optical density measured at 600 nm and **C** sugar concentration. Samples for RNA sequencing were taken at four times during the feeding phase of propagation (*t*1 = 13.5 h, *t*2 = 21.5 h, *t*3 = 29.5 h and *t*4 = 41.5 h) and are indicated in **A**. Average values and standard deviations (*n* = 3 or 4) are shown

As *S. cerevisiae* is a Crabtree-positive yeast, the presence of excess glucose during the batch phase caused a shift from complete respiratory dissimilation of glucose to a combination of respiratory and fermentative metabolism, resulting in the production of both ethanol and cell mass (Fig. 1A, B). After sugar depletion, feeding was started at a rate that allowed full respiration in non-adapting cultures. Throughout the feeding period the levels of acetic acid, furfural and HMF remained below the detection limits (data not shown). During full respiration, cell mass, which is the major product, was produced at a yield of 0.5 g_cell mass_ g_sugar_^−1^. During the feeding phase in non-adapting cultures, ethanol remaining from the batch phase was consumed and cell mass was produced (Fig. 1A, B).

The presence of hydrolysate is known to reduce respiratory capacity of *S. cerevisiae*, likely due to an increase in the requirement for cellular resources in response to inhibitor stress. As a result of this, the critical specific growth rate, i.e., the specific growth rate at which respiro-fermentative metabolism is activated, is also reduced. The cell mass yield is reduced under conditions of respiro-fermentative metabolism, as ethanol is produced as a by-product. Strains showing reduced critical specific growth rates as a consequence of genetic modifications have been reported in the literature (e.g., [31]). In the present study, it was observed that ethanol accumulated in adapting cultures during the feed phase, indicative of respiro-fermentative metabolism (Fig. 1A). The non-adapting (control) cultures showed considerably higher cell densities than adapting cultures; the OD_600_ values at the end of the feeding phase being 42.6 and 25.8, respectively (Fig. 1B), which can be explained by the difference in consumed carbon as the non-adapting cultures consumed ethanol, whereas the adapting cultures did not. The sugar consumption profiles for adapting and non-adapting propagation cultures were similar (Fig. 1C).

**Fig. 2:**
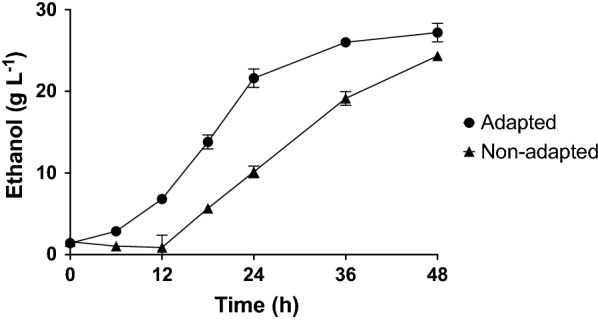
Ethanol production during fermentation of 70% (w/w) hydrolysate inoculated with adapted (circles) and non-adapted (triangles) *S. cerevisiae* CR01 cultures. Averages and standard deviations (*n* = 4) are shown

To evaluate the effect of short-term adaptation on fermentation efficiency, cell mass from hydrolysate-adapted and non-adapted cultures was harvested from the propagation cultures and used to inoculate anaerobic fermentations containing 70% (w/w) hydrolysate. Ethanol production was monitored to evaluate performance. The non-adapted yeast took 48 h to produce the same ethanol titers as the adapted yeast after 24 h (Fig. 2). These results thus confirm that short-term adaptation during propagation improves ethanol productivity during fermentation in a hydrolysate-containing medium.

### Data-driven analysis of RNA sequencing data

**Fig. 3:**
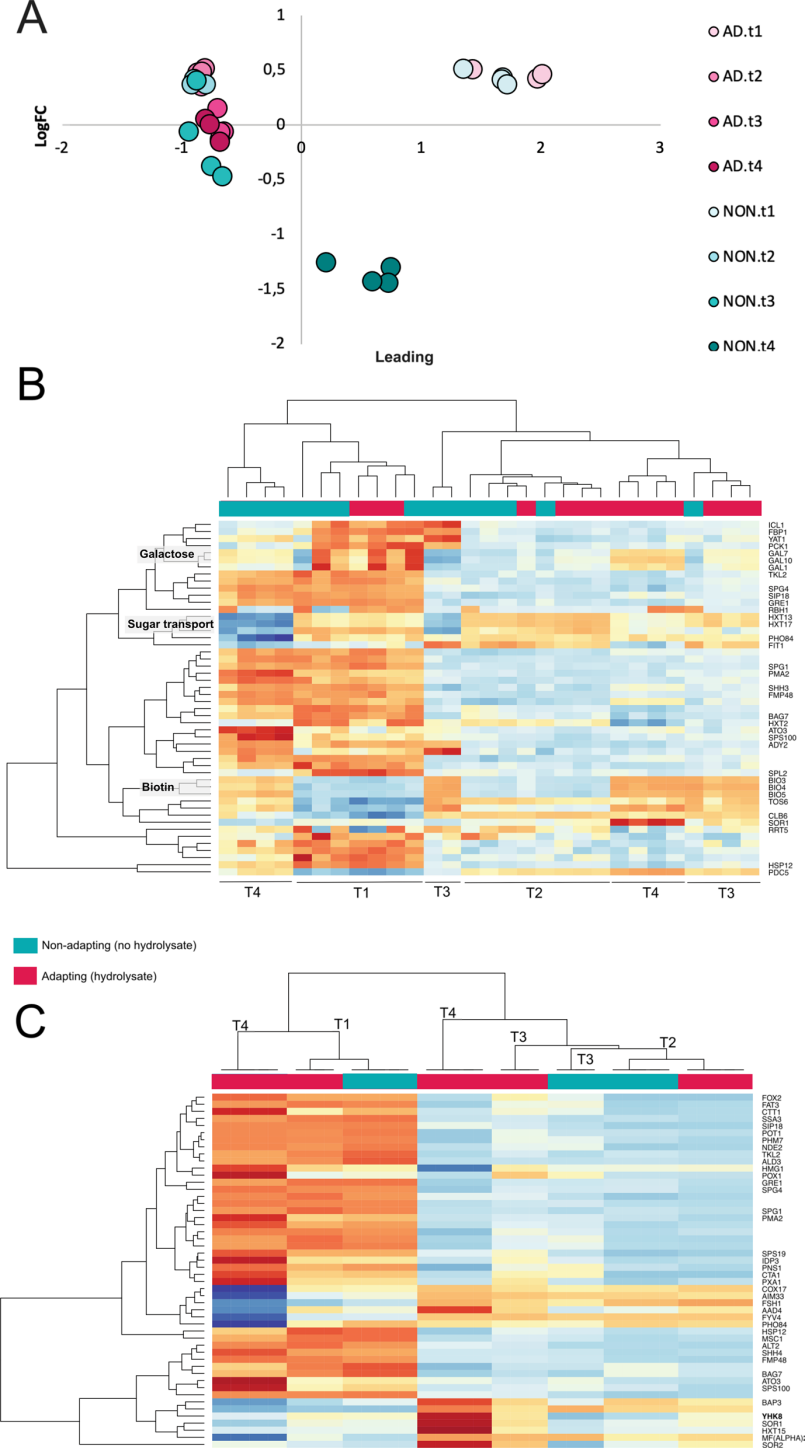
Unsupervised clustering of the RNA sequencing data showing differential expression in short-term adaptation of *S. cerevisiae* CR01 to lignocellulosic hydrolysate during aerobic propagation. Samples were taken at four times during the feeding phase of propagation (*t*1 = 13.5 h, *t*2 = 21.5 h, *t*3 = 29.5 h and *t*4 = 41.5 h). Results from all sampled biological replicates are shown (*n* = 3 or 4). **A** Multidimensional scaling of the RNA sequencing data shows major variation between t4 from adapting versus non-adapting cultures, with low variability between replicates. **B** Heatmap of the 50 most variable genes across all samples showing the differential expression during the feeding phase, including up- and down-regulation of certain biosynthetic pathway genes. **C** Heatmap of the 50 most differentially expressed genes between non-adapting and adapting cultures at the t4 time-point

To investigate the transcriptomes driving the short-term adaptation to lignocellulosic hydrolysates, total RNA from the samples collected at times t1–t4 was extracted and sequenced. One hundred base-pair paired-end reads were obtained with good sequencing quality, fulfilling the standard quality requirements set by the multiQC pipeline [32], with a high average of 24 million reads per sample (Additional file 1: Figure S1). Furthermore, the reads showed good complexity saturation (Additional file 2: Figure S2). To investigate overall trends in the data, and to further verify sample reproducibility, multivariate data analysis was performed of all the collected transcriptome data. Multidimensional scaling of all mapped reads showed that the samples grouped very precisely by time and adapting/non-adapting conditions (Fig. 3A), indicating close similarity between the replicates (*n* = 3 and 4). This data-driven analysis of the variation in the data further showed that the greatest difference in gene expression between hydrolysate-adapting and non-adapting cultures was at the last sampling point (t4, at 41.5 h). This was expected, as not only had the cells been exposed to the hydrolysate for the longest time, but also the ethanol concentrations in the two cultures were clearly different, indicating that the metabolic states of the cells at this time were very different. At the last sampling time (t4, at 41.5 h), the sample with the adapted cells still contained 15 g L^−1^ ethanol, while the non-adapted cells had already consumed all the ethanol produced (Fig. 1C). Therefore, in order to accurately identify transcriptomic trends associated with adaptation to the hydrolysate during aerobic propagation, time-resolved RNA sequencing was employed. Among the 50 most variable genes across all samples, it was seen that the transcriptomes of the non-adapting cells were similar at t1 and t4, whereas clear differences were seen in the transcriptomes of the adapted cells at t1 and t4 (Fig. 3B). The differences were even clearer when comparing the transcriptomes of the 50 most differentially expressed genes at the end of the experiments in the non-adapting and adapting cultures (Fig. 3C).

### Central carbon metabolism

To further investigate the biological relevance of the data, attention was directed to the end of propagation (t4 at 41.5 h), where the greatest differential expression was observed between adapting and non-adapting cultures. As a result of the observed respiro-fermentative metabolism in the feeding phase of hydrolysate-adapting cultures, the central carbon-metabolism pathways that start with glucose were investigated (Additional file 3: Figure S3). No significant differential expression was observed in glycolysis (p-value < 10^–4^). In the pentose-phosphate pathway, down-regulation of *TKL2* was observed in the adapting cultures, whereas *TKL1* was not differentially expressed. Tkl1 and Tkl2 are isoforms, where *TKL1* encodes the major isoform, and *TKL2* encodes the minor isoform, of transketolase [33].

In the tricarboxylic acid cycle, *MDH1, IDH1* and *IDH2* were up-regulated (Additional file 3: Figure S3)*. MDH1* encodes an NAD^+^-dependent mitochondrial malate dehydrogenase, which catalyzes the formation of oxaloacetate from malate. In addition to its role in the tricarboxylic acid cycle, Mdh1 is also a component of an NADH shuttle that regulates the NAD/NADH ratio in the mitochondria and cytoplasm [34]. *IDH1* and *IDH2* encode subunits of an NAD^+^-dependent mitochondrial isocitrate dehydrogenase complex catalyzing the oxidation of isocitrate to alpha-ketoglutarate [35]. *IDH1* overexpression has been reported to improve furfural detoxification as well as ethanol fermentation by *S. cerevisiae* [36].

In the pyruvate-to-ethanol fermentation pathway, *PDC6* and *ADH2* were found to be down-regulated, whereas *ADH3* and *ADH5* were up-regulated (p-value < 10^–4^) (Additional file 3: Figure S3). Although the pyruvate decarboxylase isoform-encoding genes *PDC1* and *PDC5* are predominantly expressed in actively fermenting cells, *PDC6* is not, but is rather actively expressed while cells are growing on non-fermentable carbon sources (Hohmann 1991). Adh2 catalyzes the conversion of ethanol to acetaldehyde [37], whereas Adh3 and Adh5 catalyze the reverse reaction [38, 39]. The down-regulation of *PDC6* and *ADH2* is thus consistent with the lack of ethanol consumption, whereas the up-regulation of *ADH3* and *ADH5* is consistent with the production of ethanol observed in the adapting cultures during the feeding phase.

### Transcription factor analysis

At the final sampling time (t4 at 41.5 h), 1162 genes were found to be differentially expressed in the adapting cultures and the non-adapting cultures (*p*-value < 10^–4^), confirming that the transcriptomes of the yeast cells were significantly different following short-term adaptation. As changes in the expression of transcription factors can sometimes provide important clues about global adaptation, the dataset at t4 was compared to the YEASTRACT transcription factor database [40].

**Fig. 4:**
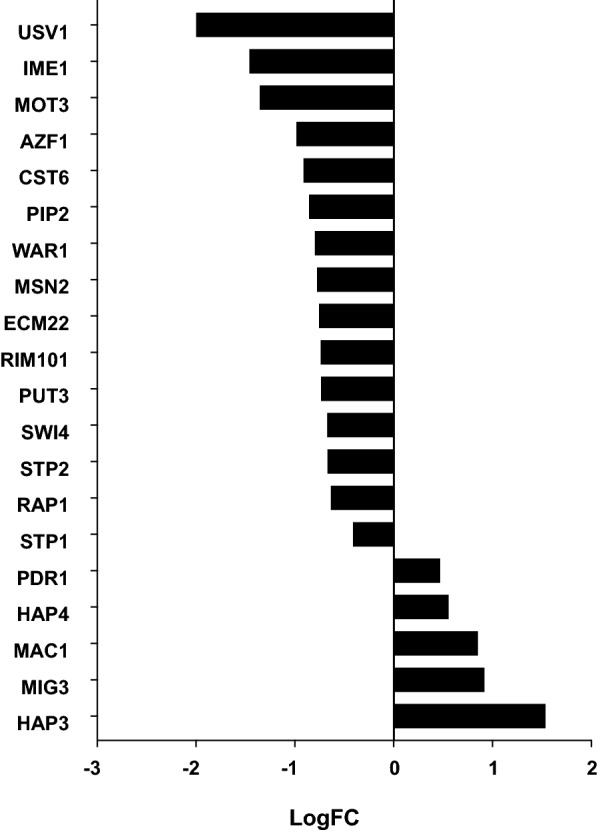
Differential gene expression at the end of short-term adaptation (t4, at 41.5 h) of *S. cerevisiae* CR01 to hydrolysate compared with non-adapting cultures (*p*-value < 10^–4^). All significantly differentially expressed transcription factors are shown. The average of biological replicates is given (*n* = 3 or 4)

Of 126 transcription factors present in the YEASTRACT database, 21 were differentially expressed in adapting compared to non-adapting cultures (p-value < 10^–4^). Of all the up-regulated transcription factors, *MIG3* is the only one reported to be involved in stress response [41] (Fig. 4). Overexpression of MIG3 has also been shown to improve ethanol tolerance, which could explain why the expression of *MIG3* was higher in the adapting cultures, where the accumulation of ethanol was observed. Many genes encoding transcription factors known to be involved in stress response were in fact down-regulated in the hydrolysate-adapting cultures, namely *USV1*, *MOT3*, *CST6*, *WAR1*, *MSN2* and *RAP1* (Fig. 4). Furthermore, genes encoding transcription factors often linked to stress response in industrial strains, such as *YAP1* and *HAA1*, were not found to be significantly differentially expressed in the adapting compared to the non-adapting cultures at t4 (*p*-value < 10^–4^). Nonetheless, transcription factor-based regulation is often dependent not only on the expression levels of the genes encoding the transcription factors, but also on the translocation of the transcription factors to the nuclei upon stress. Yap1 transits from the cytoplasm to the nucleus upon oxidative stress, and is degraded in the nucleus after the oxidative stress has passed [42, 43]. Similarly, Haa1 is phosphorylated upon acid stress, leading to its translocation into the nucleus [44].

In addition to *MIG3*, four other genes encoding transcription factors were found to be significantly up-regulated, three of which are involved in the regulation of respiratory gene expression (*HAP3*, *HAP4* and *MAC1*) and one that encodes a known regulator of multidrug resistance genes (*PDR1*, Fig. 4). Mac1 is involved in high-affinity copper transport in *S.* *cerevisiae* [45, 46]. Copper ions play an important role in the activation of the cytochrome c oxidation complex and thus in respiratory metabolism. Together with the upregulation of *HAP3* and *HAP4*, the results suggest that increased respiratory capacity could be required under adapting conditions. Pdr1 is involved in recruiting other zinc cluster proteins to pleiotropic drug response elements to fine-tune the regulation of multidrug resistance genes, as reviewed by MacPherson et al. [47]. Pdr1 has also recently been shown to be an important transcription factor involved in furfural and HMF tolerance [23].

### General stress response

The so-called general (or environmental) stress response of *S. cerevisiae* under various environmental conditions has been reported to involve ~ 900 genes, predominantly regulated by Yap1, Msn2 and Msn4 [48]. Many genes up-regulated under stressful conditions were found to be regulated by Msn2 and Msn4, which, upon stress, quickly translocates into the nucleus [49]. The expression of *MSN4* has been found to be activated by stress, whereas *MSN2* is constitutively expressed during diverse environmental stresses [48]. On the other hand, both *MSN2* and *MSN4* have been found to be highly expressed at high ethanol concentrations [50]. Msn2 and Msn4 are largely, but not completely, functionally redundant, although the regulatory contributions of Msn2/4 for specific genes may vary, depending on the particular stress condition as reviewed by Estruch [51]. Overexpression of *MSN2* has been shown to increase furfural tolerance [52]. Moreover, overexpression of a truncated form of *MSN2*, which was suggested to either modify transcriptional activity or alter the translocation of Msn2, has been shown to increase tolerance to ethanol [53, 54]. In our dataset, *MSN4* was not significantly differentially expressed in adapting cultures, whereas *MSN2* was significantly down-regulated in adapting cultures, compared to non-adapting cultures in the final sample (t4, at 41.5 h). *POX1,* encoding a protein that modulates the nucleo-cytoplasmic shuttling of *MSN2*, was also down-regulated in our dataset (Fig. 3C).

The response to short-term adaptation of genes regulated by Msn2 and Msn4 is more nuanced (Additional file 4: Table S1, Additional file 5: Table S2). A curated list of 212 open reading frames that have both expression and binding evidence for Msn2 was taken from the YEASTRACT database, as well as a list of 397 open reading frames for Msn4. From these lists, 41 genes regulated by Msn2 (Additional file 4: Table S1) and 103 genes regulated by Msn4 (Additional file 5: Table S2) were significantly differentially expressed in our dataset at t4. Some genes related to known stress responses were, as expected, up-regulated in adapting cultures. Among the genes regulated by both Msn2 and Msn4, *OYE2* and *OYE3* were among those most highly up-regulated (Additional file 4: Table S1, Additional file 5: Table S2). *OYE2* encodes an NADPH oxidoreductase, which is involved in the oxidative stress response by lowering endogenous ROS [55]. In contrast to Oye2, Oye3 has been shown to elevate cellular levels of ROS, and the formation of Oye2–Oye3 heterodimers contributes to the induction of apoptosis upon oxidative stress [55]. In our previous work, we found no significant difference in viability between adapted and non-adapted cells [11], despite the fact that many of the inhibitors found in lignocellulosic hydrolysates are known to reduce cell viability and cause apoptosis [56, 57].

A large subset of genes regulated by Msn2 and Msn4 was down-regulated at t4, *GRE1* being the most strongly down-regulated among the Msn2-regulated genes (Additional file 4: Table S1). *GRE1* and its paralog *SIP18* (also down-regulated, Fig. 3C) encode hydrophilins reported to be positively regulated by Msn2/4 and induced under stress [58], but the biological function of these genes is unknown. Among the Msn4-regulated genes, *SPG4* was the most strongly down-regulated gene, followed by *GRE1* (Additional file 5: Table S2). Spg4 is involved in the thermotolerance and longevity of stationary-phase yeast cultures [59]. In summary, cells undergoing short-term adaptation to lignocellulosic hydrolysate do not show a typical, broad stress response across a wide spectrum of genes, but rather seem to up-regulate specific genes to achieve a metabolic state that is fine-tuned for growth in the presence of lignocellulosic hydrolysate.

### Aldehyde detoxification

**Fig. 5:**
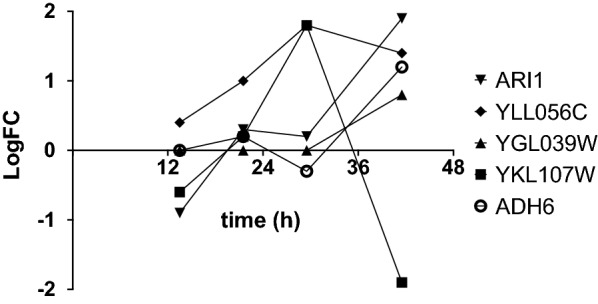
Changes in the expression of genes involved in the detoxification of furfural and hydroxymethylfurfural during short-term adaptation. Only significantly differentially expressed genes are shown (*p*-value < 10^–4^). Values given are the averages of biological replicates (*n* = 3 or 4)

In our previous work, we noted the importance of detoxification of the medium from aldehydes to allow the growth of *S. cerevisiae* [18]. Therefore, the differential expression of genes encoding oxidoreductases that have been shown to have activity on furfural and HMF was investigated. *ARI1*, *YLL056C*, *YGL039W*, *YKL107W* and *ADH6* were among the genes that were significantly up-regulated in adapting cultures, compared to non-adapting cultures (Fig. 5). The first four are known to encode furfural-converting enzymes. Adh6 has been shown to have reducing activity on HMF [24, 60]. *YLL056C* showed consistent upregulation from t2 (21.5 h) and onwards. *YLL056C* has been characterized as an NADH-dependent aldehyde reductase with confirmed activity on furfural and HMF [61]. Furthermore, both furfural and HMF were below the detection limit throughout the propagation cultures, which indicates that the cells continuously degrade these compounds during adaptation.

### Transcriptomic trends during short-term adaptation

**Fig. 6:**
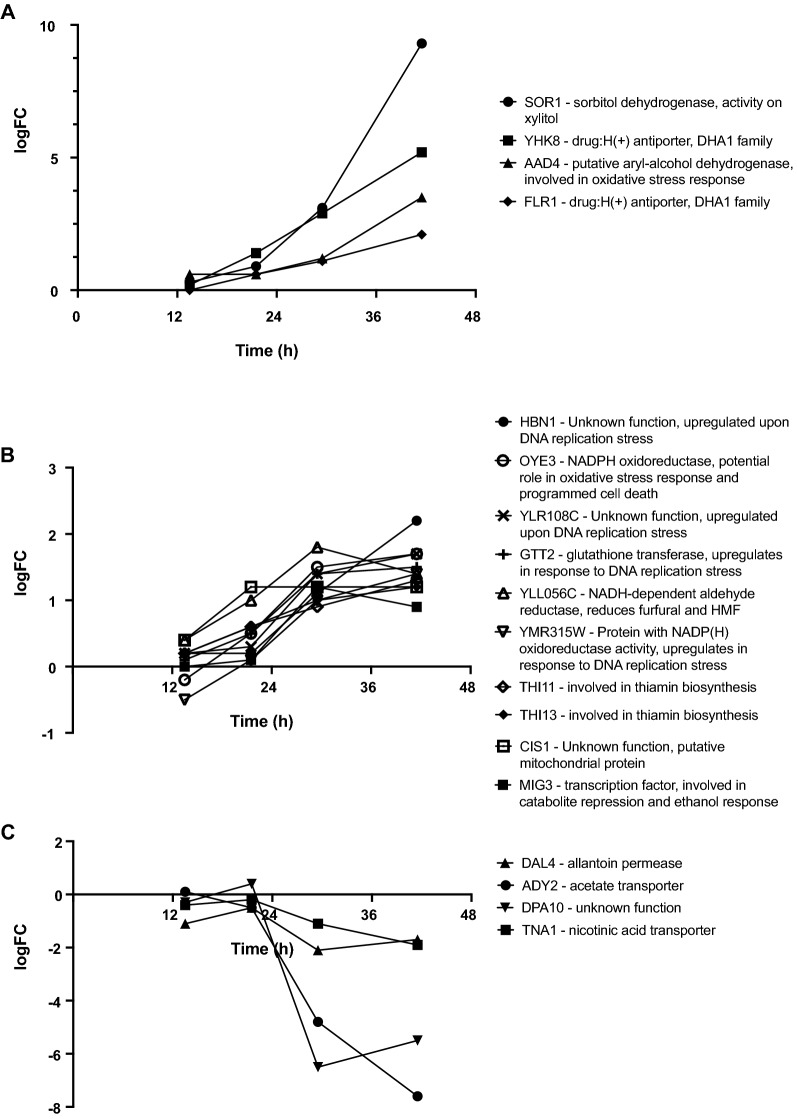
Significantly differentially expressed genes at at least 2 sampling times, showing trends of either up-regulation (**A**, **B**) or down-regulation (**C**) (*p*-value < 10^–4^). A and B are shown separately to improve visibility of genes with differential expression < 2 at the final time-point. Average values (n = 3 or 4)

The gene expression of the adapted cultures and the non-adapted cultures at t4 differed significantly in 1162 genes, while 18 genes showed a consistent trend of either up-regulation (Fig. 6A, B) or down-regulation over time (Fig. 6C, at at least two sampling times). The most highly up-regulated gene in the dataset was *SOR1* (9.3-fold up-regulated at the end of propagation). Sor1 is an NAD-dependent sorbitol dehydrogenase which is known to be induced in the presence of sorbitol or xylose, and has been shown to have some activity on xylitol [62]. Aryl-alcohol dehydrogenase Aad4 and NADPH-dependent oxidoreductase Oye3, involved in oxidative stress response and in furfural detoxification [15, 55, 63], were also up-regulated (Fig. 6A, B), in addition to *GTT2* and *YMR315W* (Fig. 6B), which encode proteins involved in DNA replication stress [64, 65]. *YLL056C* and *MIG3* (described above) are also found in this category of genes (Fig. 6B). Finally, *THI11* and *THI13,* which were also up-regulated (Fig. 6B), are genes encoding proteins involved in the synthesis of the thiamin precursor hydroxymethylpyrimidine [66]. In a previous study, we showed that supplementing non-adapting cultures with a mixture of thiamin and pyridoxine improves fermentation performance in hydrolysate-containing medium [18]. Apart from a protein of unknown function (DPA10), three out of four genes that showed a trend of down-regulation (Fig. 6C) encoded for transporters. Dal4 is an allantoin permease [67], Ady2 is an acetate transporter [68], and Tna1 is a nicotinic acid transporter [69].

### DHA1 multidrug family antiporters Yhk8 and Flr1

*YHK8* and *FLR1*, both members of the multidrug proton antiporter DHA1 family [70, 71], showed significant differential expression from t2 (21.5 h) onwards, together with an increasing trend, reaching high logFC values (Fig. 6A). While the function of Yhk8 has not been studied, the expression of *YHK8* has been shown to be up-regulated in cells exposed to azoles [72]. Flr1 has been shown to confer resistance to a number of drugs, as reviewed by Sá-Correia et al. and it has been reported that *FLR1* is induced during oxidative stress [73, 74]. The expression of *FLR1* is activated by several transcription factors, including Yap1, the main transcription factor required for oxidative stress tolerance [75]. Flr1 has been shown to confer resistance to coniferyl aldehyde, a phenolic inhibitor common in lignocellulosic hydrolysates [76]. Thus, it is plausible that Flr1 and Yhk8 could be involved in detoxification in the adapting cells, possibly through the extrusion of harmful chemicals from the cells.

**Fig. 7:**
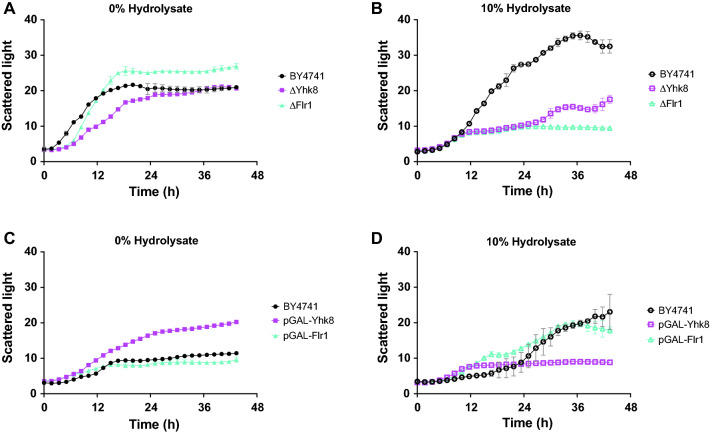
**A**, **B** Aerobic growth of BY4741, ΔYhk8 and ΔFlr1 in minimal medium supplemented with **A** sucrose or **B** sucrose + 10% wheat straw hydrolysate. **C**, **D** Aerobic growth of BY4741, BY4741 + pGAL1-YHK8 and BY4741 + pGAL1-FLR1 in minimal medium supplemented with **C** galactose or **D** galactose + 10% wheat straw hydrolysate

In order to confirm that Yhk8 and Flr1 are beneficial for growth in lignocellulosic hydrolysates, the growth of the respective deletion and overexpression mutants (in the BY4741 laboratory strain *background* acquired from the respective collections) was measured in aerobic batch cultures with varying amounts of hydrolysate (Fig. 7). Under aerobic conditions, both the ΔYhk8 and ΔFlr1 mutants showed a clear growth defect in medium supplemented with hydrolysate as the deletion strains essentially stopped growing after about 10 h, whereas the wild-type BY4741 strain continued to grow for about 36 h (Fig. 7B). In medium containing 20% hydrolysate, all the strains tested grew very poorly (data not shown). While the deletion of *YHK8* or *FLR1* clearly inhibited growth in hydrolysate-containing medium, the overexpression of either of these genes was found to reduce the lag phase of the cells during aerobic growth in medium with 10% hydrolysate compared to the wild type (Fig. 7 C and D). The biological function of Yhk8 and Flr1 during adaptation and growth in hydrolysate requires further investigation, but these transporters are clearly interesting targets for improving the performance of cells for the bioconversion of hydrolysates.

### Metabolic pathway analysis

To investigate whether metabolic pathways are transcriptionally perturbed as a result of short-term lignocellulosic adaptation, up- and down-regulated genes were analyzed in the context of their metabolic pathways (see Methods section). In particular, we evaluated the changes in fatty acid metabolism due to the potential implications on plasma membrane composition [77], as well as biotin and thiamine as we have previously shown that these improve the productivity of lignocellulosic bioethanol-producing fermentation when added to the culture during the propagation step [18].

**Fig. 8:**
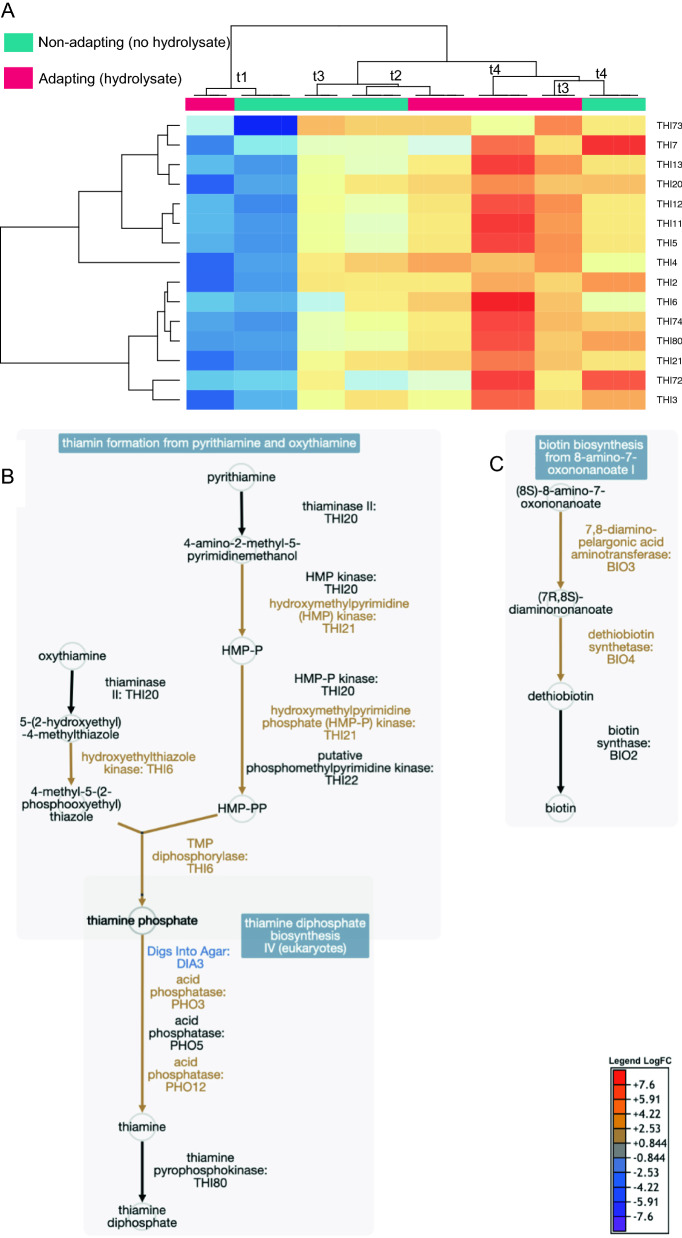
Transcriptional response of the biotin and thiamine biosynthetic pathways in hydrolysate-adapting vs. non-adapting propagation cultures, showing up-regulation of both pathways over time (time points t1–t4). **A** Clustered thiamine biosynthetic genes were up-regulated during propagation (red shades denote up-regulation, and blue shades down-regulation). The results for all biological replicates are shown (*n* = 3 or 4). **B** Thiamine biosynthetic pathway genes were significantly (*p*-value < 10^–4^) up-regulated at the end of adapting vs. non-adapting cultivations (indicated by colored reaction arrows and text). **C** Biotin biosynthetic pathway genes were significantly up-regulated (*p*-value < 10^–4^) at the end of adapting vs. non-adapting cultivations (indicated by colored reaction arrows and text)

The three biotin biosynthetic genes *BIO3, BIO4* and *BIO5* were gradually up-regulated during both non-adapting and adapting cultivations (Fig. 3B). At the end of propagation, *BIO3* and *BIO4* were significantly more up-regulated in the adapting cultures. As Bio3 and Bio4 catalyze the two committed reactions from biotin from 8-amino-7-oxononanoate to dethiobiotin, they are likely to be rate-limiting enzymes in biotin biosynthesis in *S. cerevisiae*. Endogenous production may not provide sufficient biotin during growth in the absence of biotin in the medium [78]. In contrast, *BIO2,* which encodes the final biotin synthase, was not differentially expressed compared to the non-adapted cultures, although it was up-regulated at the end of propagation compared to t1 (13.5 h) (Fig. 8). The hydrolysate-triggered up-regulation of *BIO3* and *BIO4* suggests that cells naturally respond to the presence of hydrolysate inhibitors by further increasing their requirement for biotin. This casts new light on previous results showing improved ethanol productivity (and maintained cell viability) in hydrolysates when propagating yeasts supplemented with biotin [8, 79, 18]. In the present study, we found that biotin biosynthesis genes were up-regulated during both hydrolysate-adapting and non-adapting cultures, probably as the demand for this stress-protective nutrient increases during propagation.

Most genes involved in the thiamine diphosphate biosynthetic pathway were up-regulated during propagation, under both adapting and non-adapting conditions (Fig. 8A). This may reflect the fact that thiamine plays an important role in central carbon metabolism by binding to pyruvate decarboxylase Pdc1 [80] and pyruvate dehydrogenase Pda1 [81]. Thiamine diphosphate also plays an important role in protection against oxidative stress [82]. Thiamine diphosphate binds to the transketolases that are used for xylose consumption and in the pentose-phosphate pathway, which is important in providing NADPH during oxidative stress such as that resulting from lignocellulosic inhibitors. The thiamine biosynthetic genes *THI6* and *THI13* were further up-regulated in the hydrolysate-adapting cultures (p-value < 10^–4^), although most other *THI* genes were also up-regulated to some extent (Fig. 8A). This indicates that xylose-consuming *S. cerevisiae* naturally responds to short-term adapting conditions by increasing its requirement for thiamine diphosphate and biotin.

## Conclusions

Transcriptional profiles were compared during the course of adapting and non-adapting propagation. Short-term adaptation of *S.* *cerevisiae* to wheat straw hydrolysate during propagation led to significant transcriptional changes. Oxidative stress response genes appear to be important, as does the up-regulation of genes encoding detoxifying enzymes acting on furaldehydes. Biotin and thiamine metabolism were found to be of particular interest, as nutrient conditions in lignocellulose hydrolysates are often poor. Yhk8 and Flr1, belonging to the DHA1 multidrug proton antiporter family, were identified as targets for future research as they showed strong up-regulation throughout the short-term adaptation process.

## Materials and methods

### Microorganisms and cultivation

The industrial strain of *S. cerevisiae* used for RNA sequencing in this study, CR01, was kindly provided by Taurus Energy AB, Sweden. The strain has been genetically and evolutionary engineered to harbor xylose fermentation capability and to ferment efficiently in the presence of lignocellulose inhibitors. It was stored at − 80 °C in a 30% (w/w) glycerol solution.

#### Seed cultivation

Before propagation, the frozen cell stock solution was thawed and grown for 24 h in synthetic minimal medium containing 20 g L^−1^ glucose at pH 6.0. Other components were added according to Verduyn et al. [83] with the exception of ammonium sulfate, which was replaced with 2.3 g L^−1^ urea to prevent acidification of the medium. Incubation was performed at 30 °C on an orbital shaker (IKA, Germany) at 200 rpm (orbital diameter: 20 mm) in 250-mL shake flasks with a working volume of 50 mL.

#### Propagation

Aerobic, fed-batch propagation was performed in 3.6-L bioreactors (Infors, Switzerland). The batch medium was made using molasses which was diluted to a final sucrose concentration of 20 g L^−1^ and sterilized using 0.2-µm nylon membrane filters. In addition to molasses, the batch medium contained the following compounds: 5 g L^−1^ ammonium sulfate, 3 g L^−1^ potassium phosphate, 0.5 g L^−1^ magnesium sulfate, 0.033 mg L^−1^ D-biotin, and 0.1 g L^−1^ polypropylene glycol 2000. The batch cultivations, with a working volume of 0.5 L, were maintained at 30 °C, and at pH 5.0 by the addition of 2 M potassium hydroxide solution throughout propagation. A cascade control was triggered when the dissolved oxygen in the reactor decreased below 40%. Agitation was gradually increased from 800 to 1000 rpm, and the air flow into the reactor from 1 to 2 vvm. After the sucrose in the batch medium had been depleted, the feed was controlled so as to maintain a specific growth rate of 0.05 h^−1^. All feed solutions contained a total concentration of 100 g L^−1^ sucrose and/or glucose, and 14 g L^−1^ xylose. In order to adapt the yeast, a feed solution was prepared using 40% (w/w) WSH and molasses to total 100 g L^−1^ sucrose and/or glucose, and 14 g L^−1^ xylose. The composition of the hydrolysate was 64.9 g L^−1^ glucose, 23.8 g L^−1^ xylose, 1.0 g L^−1^ formic acid, 5.1 g L^−1^ acetic acid, 0.3 g L^−1^ HMF and 2.2 g L^−1^ furfural. A reference solution was also prepared consisting of sucrose from beet molasses with 14 g L^−1^ xylose. Both the feed and the reference solutions were supplemented with 28 g L^−1^ ammonium sulfate, 3 g L^−1^ potassium phosphate, 0.5 g L^−1^ magnesium sulfate, 0.033 mg L^−1^ D-biotin, and 0.1 g L^−1^ polypropylene glycol 2000. The feed solutions were pumped into the reactor at an exponentially increasing rate over a period of 28 h. Samples were collected for RNA sequencing analysis throughout the feeding phase (Fig. 1). The protocol for sample handling and RNA extraction is described in the section ‘RNA extraction’. After propagation, the cells were evaluated for fermentation performance. To this extent, cells were harvested by centrifugation (3800 × g, 5 min), followed by washing with 9 g L^−1^ sterile sodium chloride solution. The cell pellets were then resuspended in the fermentation medium and used to inoculate fermentation cultures.

#### Fermentation

Batch fermentation was performed in 500-mL screw-top shake flasks (Duran, Germany) with a one-way valve connected to the cap (Eppendorf, Germany) to allow for carbon dioxide release. Another connection allowed for sterile sampling through a swabable valve. The working volume for fermentation was 200 mL. The fermentation medium contained 70% (w/w) WSH supplemented with 2.3 g L^−1^ urea, 3 g L^−1^ potassium phosphate, 0.5 g L^−1^ magnesium sulfate, and 0.033 mg L^−1^ D biotin. The fermentation broth was inoculated with 1 g L^−1^ cell dry weight (CDW), and incubated on an orbital shaker at 150 rpm, at 30 °C, for 48 h. The weight loss (due to carbon dioxide release) was monitored and used to determine the progress of fermentation over time.

#### Follow-up studies

*S. cerevisiae* BY4741 (MATa *his3*Δ1 *leu2*Δ0 *ura3*Δ0 *met15*Δ0 – s288c background), the BY4741-derived and ΔYhk8 and ΔFlr18 mutants included in the EUROSCARF collection, as well as the pGAL-YHK and pGAL-FLR1 overexpression strains (kept in BY4741 through uracil selection) of the Yeast GST Fusion collection (available through Horizon Discovery) were used for studies in microbioreactors to follow-up RNA sequencing results (Biolector, Bio2labs) as described by van Dijk et al. [84]. Pre-cultures for these follow-up studies were grown in minimal medium [83] in which ammonium sulfate was replaced with 2.3 g L^−1^ urea, either 20 g L^−1^ sucrose (for deletion strains) or 20 g L^−1^ galactose (for overexpression strains) was used as a carbon source and either 0.79 g L^−1^ Complete Supplement Mixture (CSM; mpbio, USA) or 0.77 g L^−1^ CSM-urea were added. Varying concentrations of WSH were added to the media to evaluate the growth in hydrolysate. Aerobic cultures containing 0, 10 or 20% WSH were inoculated at an initial optical density (OD_600nm_) of 0.5, whereas anaerobic cultures with 30 or 50% WSH were inoculated at a starting OD_600nm_ of 1. Uracil selection was used for plasmid maintenance in aerobic cultivations of overexpression strains, but not for the anaerobic cultures, where the high amounts of WSH ensure uracil availability. All cultivations were performed in duplicate, some in triplicate.

### Analytical methods

#### Cell density measurements

The OD_600nm_ was determined by measuring the absorbance of the cell culture at a wavelength of 600 nm using a Genesys 20 spectrophotometer (Thermo Scientific, USA). The OD_600nm_ obtained from filtered samples was subtracted to compensate for the background color of the medium. CDW was determined by filtering appropriate volumes (containing a minimum of 10 mg CDW and a maximum of 40 mg CDW on the filter) of cell culture through a pre-dried and weighed 0.45 µm polyethersulfone membrane (Sartorius, Germany). The filters containing samples were washed with deionized water and dried using a microwave oven at a power output of 385 W for 15 min, before weighing.

#### Metabolite and inhibitor analysis

The concentrations of extracellular metabolites, sugars and inhibitors were determined by HPLC, using a refractive index detector (Jasco, Italy). Measurements were performed on filtered samples (0.2-µm nylon membrane filters, VWR, USA). Glucose, xylose, arabinose, formic acid, acetic acid, HMF, and furfural were separated using a Rezex ROA-organic acid H + column at a flow rate of 0.8 mL min^−1^, at 80 °C, using 5 mM sulfuric acid solution as eluent. Sucrose, fructose, mannose, and galactose were separated using a Rezex RPM Monosaccharide Pb + column at a flow rate of 0.6 mL min^−1^, at 85 °C, using Milli-Q water as eluent. Both columns were purchased from Phenomenex (USA).

### Feed rate

The feed rate [L h^−1^] for propagation was calculated using the following equation:

$$ F\left( t \right) = \frac{{\mu_{s} S_{i} V\left( {t_{0} } \right)}}{{S_{F} }}\exp \left( {\mu_{s} t} \right), $$

where $\mu_{s}$ is the desired, constant specific growth rate during the feed phase [h^−1^], $S_{i}$ is the concentration of sucrose at the start of the batch phase [g L^−1^], $V\left( {t_{0} } \right)$ is the working volume of the culture when starting the feed [L], $S_{F}$ is the concentration of sucrose in the feed solution [g L^−1^], and $t$ is the time that has expired since starting the feed.

### RNA sequencing

Library preparation and sequencing were performed at the SNP&SEQ Technology Platform (Uppsala, Sweden) using the TruSeq Stranded mRNA-seq protocol and the NovaSeq 6000 system, respectively.

#### RNA extraction

RNA was extracted from cells harvested during the feeding phase of exponentially fed cultivations. Four samples were collected at different times denoted t1–t4, where t1 is the time at which feeding was started, and t4 the time when feeding was stopped (t1 + 28 h). The control cultivations (non-adapting) were fed with a solution containing 100 g L^−1^ glucose and 14 g L^−1^ xylose, whereas the adapted cultivations were fed with a 40% (w/w) WSH solution, supplemented with glucose to obtain the same concentrations of fermentable sugars in both feed solutions. Samples for RNA sequencing were directly deposited in pre-cooled conical tubes, which were kept in a 50% ethanol–ice solution during sample preparation. Cells were washed using a 9 g L^−1^ sodium chloride solution that was cooled to 4 °C and kept on ice. Cells were centrifuged at 0 °C at 3800 × g. After washing, the cell pellet was resuspended in RNAlater (Invitrogen, USA) and kept at -20 °C until analyzed. RNA was extracted using a TRIzol–chloroform method, as described by Geijer et al. [85], followed by on-column DNase digestion using the RNeasy PowerPlant Kit and RNase-free DNAse set from Qiagen (Germany). The RNA samples were analyzed with a Fragment Analyzer (Agilent, USA) and all samples were confirmed to have an RNA integrity number above 5.5.

#### Library preparation

Sequencing libraries were prepared from 1 μg total RNA using the TruSeq stranded mRNA library preparation kit (cat# 20020595, Illumina Inc., USA) including polyA selection. Unique dual indexes (cat# 20022371, Illumina Inc., USA) were used. Library preparation was performed according to the manufacturer’s protocol (#1000000040498).

#### Sequencing

The libraries were sequenced using a NovaSeq 6000 system (Illumina Inc., USA) with paired-end 100-bp read length and v1 sequencing chemistry. A sequencing library for the phage PhiX was included as a 1% spike-in in the sequencing run. The sequencing yielded a coverage of 17 to 35 M reads per library.

#### Data quality control

Quality control for sequencing reads was performed using FastQC v0.11.8 [86]. Duplication rates for genes were analyzed using dupRadar v1.14.0 [87]. The complexity of the libraries was estimated using Preseq v2.0.3 [88]. Several modules of the RSeQC package v3.0.1 [89] were used, such as ‘read distribution’, ‘inner distance’, ‘read duplication’, ‘junction saturation’ and ‘infer experiment’. Alignments were assembled into potential transcripts using StringTie v2.0 [90], giving Fragments Per Kilobase of transcript per Million mapped reads (FPKM) metrics for genes and transcripts as outputs. Analysis of adapter contamination and trimming of low-quality regions was performed with TrimGalore v0.6.4 [91]. Reads were mapped to reference genome R64-1-1 (*S. cerevisiae*) using the STAR alignment tool vSTAR_2.6.1d [92]. The Subread package featureCounts v1.6.4 was used to obtain counts of reads mapping to genes [93]. The quality control output files were visualized using MultiQC [32].

#### Differential gene expression analysis

Gene counts obtained by using featureCounts were imported into R, all subsequent differential gene expression analysis was performed using the R package EdgeR [94]. Genes with low expression were filtered out of the gene counts using the ‘filterByExpr’ function. Library sizes were then normalized using scaling factors using a trimmed mean of M-values between each pair of the samples calculated by the ‘calcNormFactors’ function. This normalization minimizes the values of log-fold change (logFC) between the samples for most genes. Common and tagwise dispersions were estimated using the ‘estimateDisp’ function. Differential expression was determined by performing quasi-likelihood F-tests (QLF test) using the ‘glmQLFtest’ function. QLF tests are generally preferred over likelihood ratio tests as the former reflect the uncertainty in estimating the differential expression for each gene. The Omics Dashboard was used to explore and visualize the gene expression data [95].

## Supplementary Information

**Additional file 1: Figure S1**. FastQC quality scores for the sequencing libraries used in this study.

**Additional file 2: Figure S2**. PreSEQ complexity curves for the sequencing libraries involved in this study.

**Additional file 3: Figure S3**. Differential expression of genes involved in central carbon metabolism in adapting cultures compared to non-adapting cultures at t4 (41.5 h). (Only results with a p-value < 10-4 are shown). Values given are the average of biological replicates (n = 3 or 4). Black arrows and text indicate genes that were not found to be significantly differentially expressed. Colored arrows and text indicate the value of logFC with which the corresponding genes were differentially expressed, according to the legend in the bottom right-hand corner of the figure.

**Additional file 4: Table S1**. Differential expression of genes regulated by Msn2 as listed in the YEASTRACT database at the end of short-term adaptation (41.5 h) when comparing adapting to non-adapting cultures of CR01. Only results with a p-value < 10-4 are shown. Values given are the average of biological replicates (n = 3 or 4).

**Additional file 5: Table S2**. Differential expression of genes regulated by Msn4 as listed in the YEASTRACT database at the end of short-term adaptation (41.5 h) when comparing adapting to non-adapting cultures of CR01. Only results with a p-value < 10-4 are shown. Values given are the average of biological replicates (n = 3 or 4).

## Data Availability

The dataset(s) supporting the conclusions of this article is(are) included within the article (and its additional file(s)).
