# Supplementary figures and images for: RNA sequencing reveals metabolic and regulatory changes leading to more robust fermentation performance during short-term adaptation of Saccharomyces cerevisiae to lignocellulosic inhibitors
Source: Biotechnol Biofuels. 2021 Oct 15;14:201. doi: 10.1186/s13068-021-02049-y (PMC8518171; doi:10.1186/s13068-021-02049-y)

FastQC: Mean Quality Scores

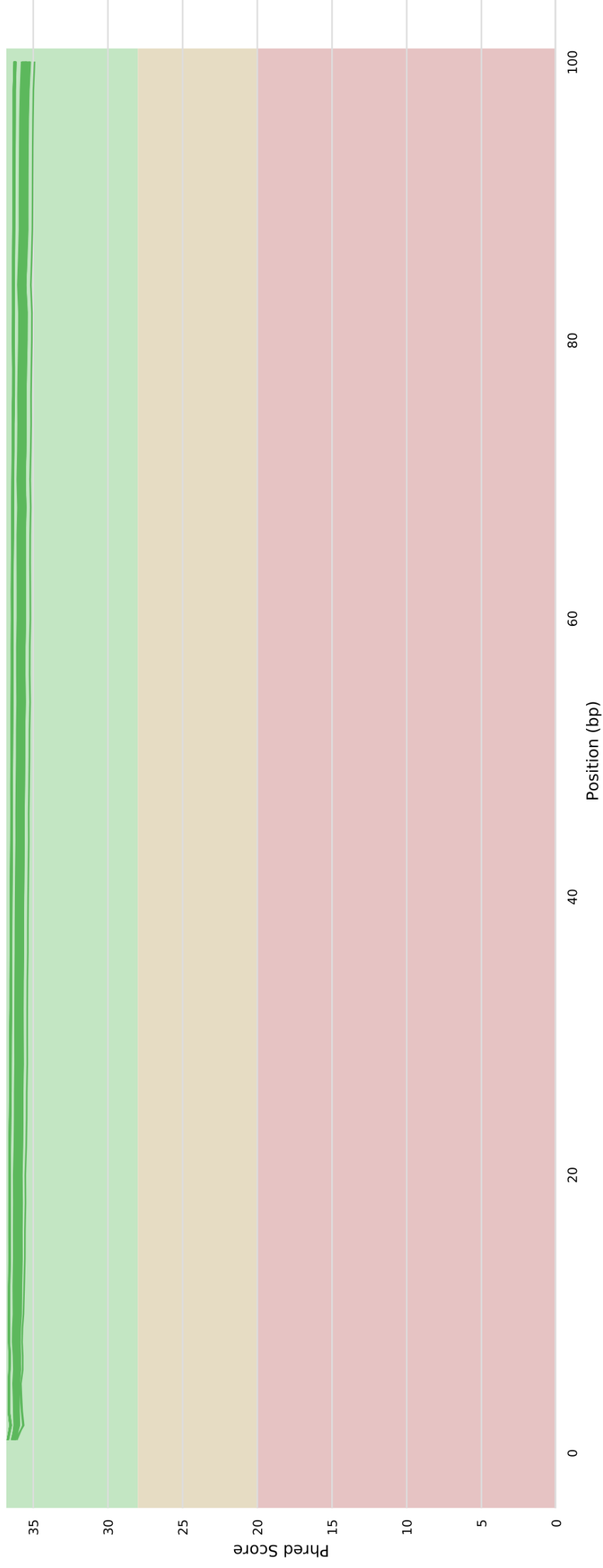

Supplement: Supplementary file 1 — Additional file 1: Figure S1. FastQC quality scores for the sequencing libraries used in this study. [file 13068_2021_2049_MOESM1_ESM.pdf]

Preseq: Complexity curve

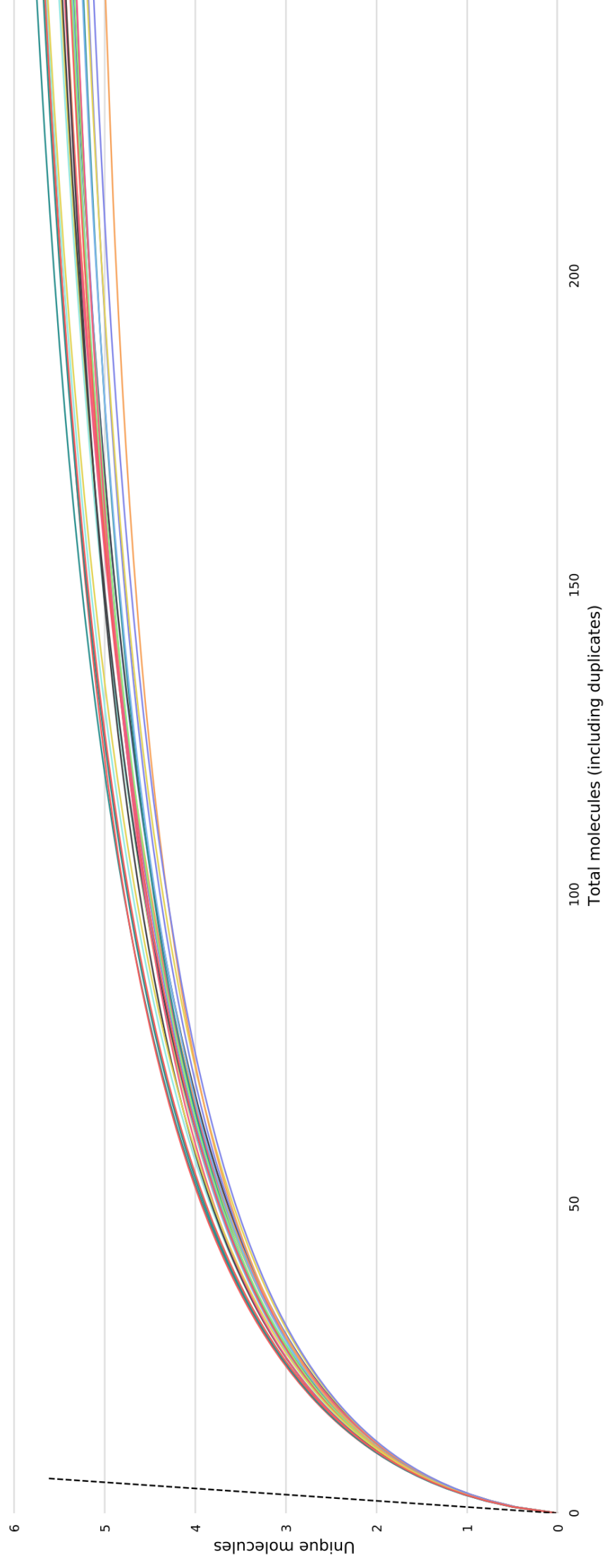

Supplement: Supplementary file 2 — Additional file 2: Figure S2. PreSEQ complexity curves for the sequencing libraries involved in this study. [file 13068_2021_2049_MOESM2_ESM.pdf]

glycolysis III (from glucose)

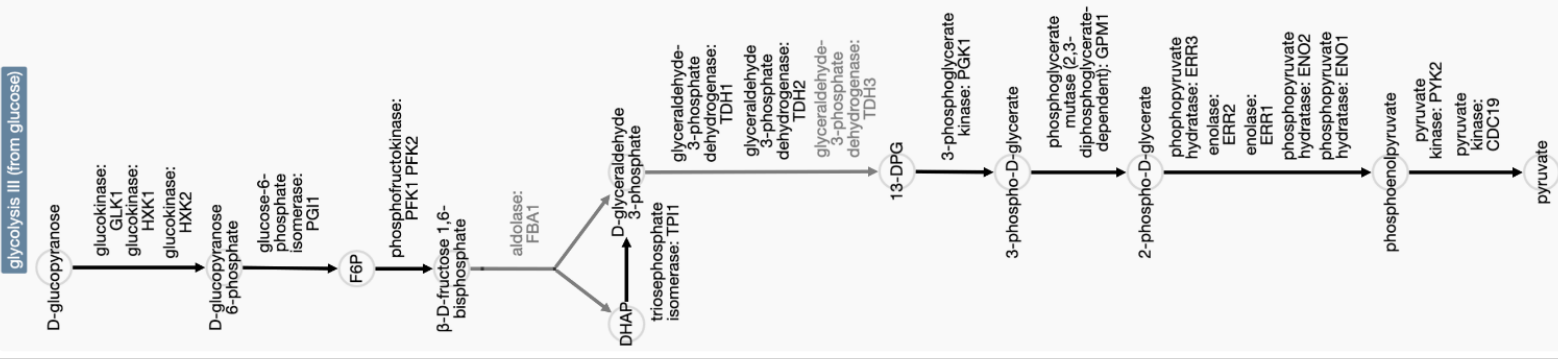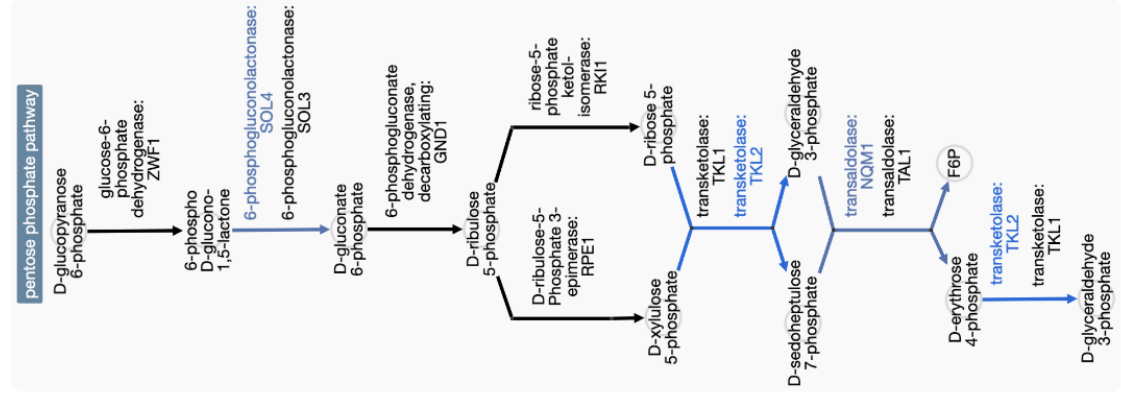

TCA cycle II (plants and fungi)

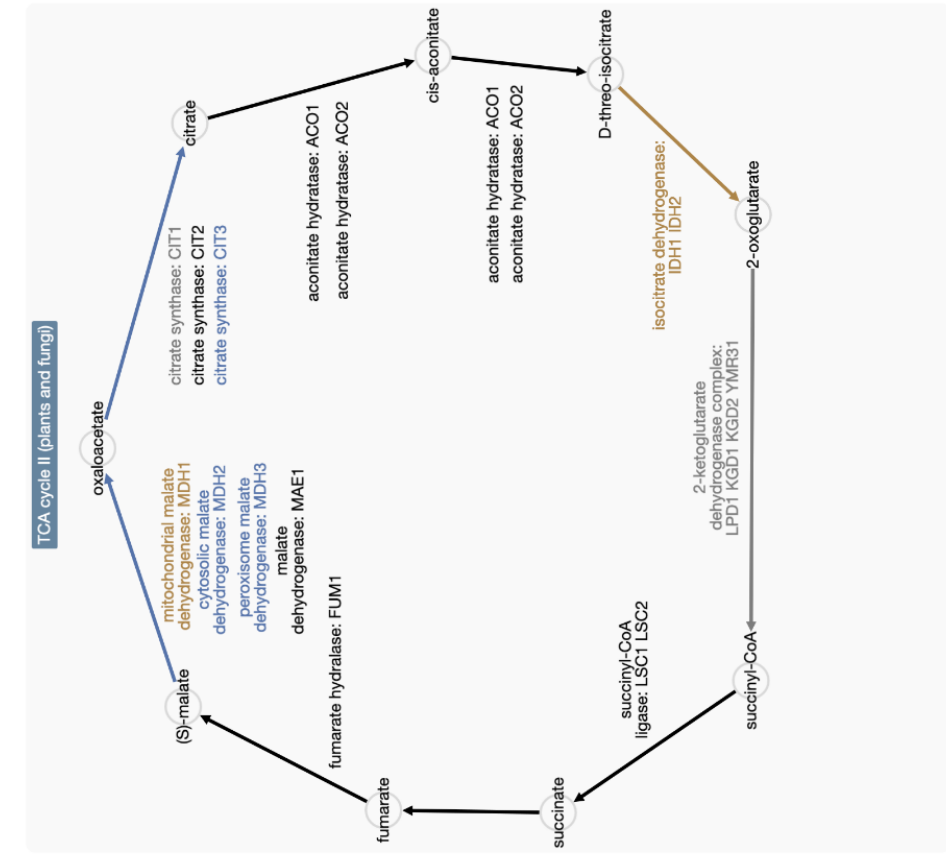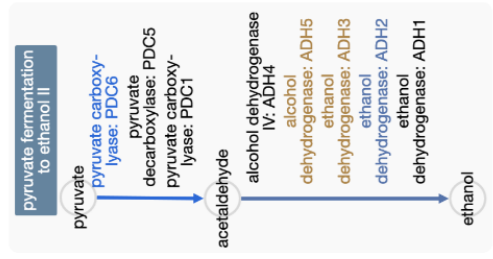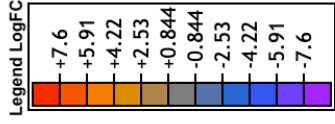

Supplement: Supplementary file 3 — Additional file 3: Figure S3. Differential expression of genes involved in central carbon metabolism in adapting cultures compared to non-adapting cultures at t4 (41.5 h). (Only results with a p-value < 10-4 are shown). Values given are the average of biological replicates (n = 3 or 4). Black arrows and text indicate genes that were not found to be significantly differentially expressed. Colored arrows and text indicate the value of logFC with which the corresponding genes were differentially expressed, according to the legend in the bottom right-hand corner of the figure. [file 13068_2021_2049_MOESM3_ESM.pdf]
