# Supplementary material for: RNA sequencing reveals metabolic and regulatory changes leading to more robust fermentation performance during short-term adaptation of Saccharomyces cerevisiae to lignocellulosic inhibitors
Source: Biotechnol Biofuels. 2021 Oct 15;14:201. doi: 10.1186/s13068-021-02049-y (PMC8518171; doi:10.1186/s13068-021-02049-y)
Supplement: Supplementary file 4 — Additional file 4: Table S1. Differential expression of genes regulated by Msn2 as listed in the YEASTRACT database at the end of short-term adaptation (41.5 h) when comparing adapting to non-adapting cultures of CR01. Only results with a p-value < 10-4 are shown. Values given are the average of biological replicates (n = 3 or 4). [file 13068_2021_2049_MOESM4_ESM.docx]

Table A1. Differential expression of genes regulated by Msn2 as listed in the YEASTRACT database at the end of short-term adaptation (41.5 h) when comparing adapting to non-adapting cultures of CR01. Only results with a p-value < 10^-4^ are shown. Values given are the average of biological replicates (n = 3 or 4).

| Gene name | LogFC |
| --- | --- |
| GRE1 | -6.6 |
| CTA1 | -4.1 |
| SHH4 | -3.3 |
| SSA3 | -3.3 |
| ALT2 | -2.7 |
| CIT3 | -2.3 |
| RGI2 | -2.1 |
| PDR10 | -1.7 |
| ERG25 | -1.3 |
| AAC1 | -1.3 |
| TIP1 | -1.2 |
| TES1 | -0.9 |
| FBA1 | -0.8 |
| PEX28 | -0.8 |
| MUB1 | -0.7 |
| EPL1 | -0.6 |
| VTC3 | 0.5 |
| MCT1 | 0.6 |
| YGL039W | 0.8 |
| ERP2 | 0.8 |
| CYC1 | 0.9 |
| CCS1 | 0.9 |
| MCP2 | 1.0 |
| TOH1 | 1.1 |
| BIO3 | 1.1 |
| AHA1 | 1.1 |
| HEM12 | 1.1 |
| CBP3 | 1.2 |
| MAS1 | 1.2 |
| HUG1 | 1.3 |
| KTR2 | 1.3 |
| MSS51 | 1.3 |
| HCH1 | 1.3 |
| PPN2 | 1.4 |
| RMI1 | 1.4 |
| AGA2 | 1.5 |
| PHO12 | 1.6 |
| OYE3 | 1.7 |
| HXT8 | 1.9 |
| MRPL3 | 1.9 |
| OYE2 | 3.6 |
