# Supplementary material for: RNA sequencing reveals metabolic and regulatory changes leading to more robust fermentation performance during short-term adaptation of Saccharomyces cerevisiae to lignocellulosic inhibitors
Source: Biotechnol Biofuels. 2021 Oct 15;14:201. doi: 10.1186/s13068-021-02049-y (PMC8518171; doi:10.1186/s13068-021-02049-y)
Supplement: Supplementary file 5 — Additional file 5: Table S2. Differential expression of genes regulated by Msn4 as listed in the YEASTRACT database at the end of short-term adaptation (41.5 h) when comparing adapting to non-adapting cultures of CR01. Only results with a p-value < 10-4 are shown. Values given are the average of biological replicates (n = 3 or 4). [file 13068_2021_2049_MOESM5_ESM.docx]

Table A2. Differential expression of genes regulated by Msn4 as listed in the YEASTRACT database at the end of short-term adaptation (41.5 h) when comparing adapting to non-adapting cultures of CR01. Only results with a p-value < 10^-4^ are shown. Values given are the average of biological replicates (n = 3 or 4).

| Gene name | LogFC |
| --- | --- |
| SPG4 | -7.9 |
| GRE1 | -6.6 |
| SPS100 | -5.7 |
| CTT1 | -4.7 |
| PUT4 | -4.2 |
| YBR116C | -4.1 |
| HSP12 | -4.0 |
| FMP48 | -3.9 |
| YNL194C | -3.7 |
| SIP18 | -3.6 |
| SHH4 | -3.3 |
| PHM7 | -3.3 |
| SSA3 | -3.3 |
| HBT1 | -3.2 |
| PDC6 | -3.1 |
| ALD4 | -2.9 |
| POX1 | -2.9 |
| DDR2 | -2.8 |
| SUL1 | -2.8 |
| HXT5 | -2.7 |
| YJR115W | -2.7 |
| FMP45 | -2.6 |
| SDH9 | -2.4 |
| MDH2 | -2.4 |
| PAI3 | -2.3 |
| CYB2 | -2.3 |
| SUE1 | -2.2 |
| LSP1 | -2.1 |
| RGI2 | -2.1 |
| GIP2 | -2.1 |
| USV1 | -2.0 |
| EIS1 | -1.9 |
| OLE1 | -1.9 |
| SFK1 | -1.8 |
| NCE102 | -1.8 |
| UBX6 | -1.8 |
| IDP2 | -1.8 |
| RTN2 | -1.8 |
| FAA1 | -1.8 |
| GLT1 | -1.7 |
| TMA10 | -1.7 |
| PDR10 | -1.7 |
| DDI3 | -1.7 |
| PIL1 | -1.6 |
| PRR2 | -1.5 |
| DCS2 | -1.5 |
| RTC3 | -1.5 |
| TDA1 | -1.4 |
| MOT3 | -1.4 |
| YIR016W | -1.3 |
| SGA1 | -1.3 |
| SPI1 | -1.2 |
| TPS2 | -1.2 |
| TIP1 | -1.2 |
| SUR1 | -1.0 |
| HPT1 | -0.9 |
| IRC24 | -0.9 |
| DIA3 | -0.9 |
| MSN2 | -0.8 |
| HSP42 | -0.8 |
| ADE17 | -0.7 |
| PUT3 | -0.7 |
| KNS1 | -0.7 |
| RNY1 | -0.7 |
| PFK26 | -0.6 |
| WTM1 | -0.6 |
| SNF6 | -0.6 |
| NUP120 | -0.6 |
| HAP4 | 0.6 |
| UFD1 | 0.6 |
| AFG1 | 0.6 |
| HSP78 | 0.7 |
| VMA13 | 0.7 |
| CIT1 | 0.8 |
| CBP2 | 0.9 |
| MDH1 | 0.9 |
| LEU2 | 1.0 |
| IDH1 | 1.0 |
| ATP7 | 1.0 |
| YBR284W | 1.1 |
| MRPL16 | 1.1 |
| NEL1 | 1.1 |
| AHA1 | 1.1 |
| PET123 | 1.2 |
| RPL9B | 1.2 |
| MRPL22 | 1.2 |
| KTR2 | 1.3 |
| RSM10 | 1.3 |
| MRPS17 | 1.4 |
| YMR315W | 1.4 |
| MDJ1 | 1.5 |
| OYE3 | 1.7 |
| HXT8 | 1.9 |
| YHR033W | 2.2 |
| GUP2 | 2.2 |
| TIR1 | 2.2 |
| ARO10 | 3.3 |
| OYE2 | 3.6 |
| HXT16 | 3.9 |
| HXT15 | 4.2 |
| HXT13 | 5.0 |
| YHK8 | 5.2 |
| SOR1 | 9.3 |
